# Native nephrectomy prior to pediatric kidney transplantation: biological and clinical aspects

**DOI:** 10.1007/s00467-012-2115-y

**Published:** 2012-02-26

**Authors:** Fatemeh Ghane Sharbaf, Martin Bitzan, Konrad M. Szymanski, Lorraine E. Bell, Indra Gupta, Jean Tchervenkov, John-Paul Capolicchio

**Affiliations:** 1 Division of Nephrology, Montreal Children’s Hospital and McGill University, 2300, rue Tupper—E222, Montreal, Quebec Canada H3H 1P3; 2 Department of Pediatrics, Dr. Sheikh Hospital, Mashhad University of Medical Sciences, Mashhad, Islamic Republic of Iran; 3 Division of Urology, Montreal Children’s Hospital and McGill University, Montreal, Quebec Canada; 4 Department of Surgery and Multiorgan Transplant Program, Royal Victoria Hospital and McGill University, Montreal, Quebec Canada

**Keywords:** CAKUT, Cystinosis, Nephrotic syndrome, Peritoneal dialysis, Polyuria, Proteinuria, Retroperitoneoscopic nephrectomy

## Abstract

**Background:**

Pre-transplant nephrectomy is performed to reduce risks to graft and recipient. The aims of this study were to evaluate (1) indications, surgical approach, and morbidity of native nephrectomy and (2) the effects of kidney removal on clinical and biological parameters.

**Methods:**

This study was designed as a single-center retrospective cohort study in which 49 consecutive patients with uni- or bilateral native nephrectomies were identified from a total of 126 consecutive graft recipients in our pediatric kidney transplantation database between 1992 and 2011. Demographic, clinical, and laboratory details were extracted from charts and electronic records, including operation reports and pre- and post-operative clinic notes.

**Results:**

Of the 49 nephrectomized patients, 47% had anomalies of the kidneys and urinary tract, 22% had cystinosis, 12% had focal segmental glomerulosclerosis, and 6% had congenital nephrotic syndrome. Nephrectomy decisions were based on clinical judgment, taking physiological and psychosocial aspects into consideration. Nephrectomy was performed in patients with polyuria (>2.5 ml/kg/h) and/or large proteinuria (>40 mg/m^2^/h), recurrent urinary tract infection or (rarely) hypertension. Urine output decreased from (median) 3.79 to 2.32 ml/kg/h (−34%), and proteinuria from 157 to 100 mg/m^2^/h (−40%) after unilateral nephrectomy (*p* = 0.005). After bilateral nephrectomy, serum albumin, protein and fibrinogen concentrations normalized in 93, 73, and 55% of nephrectomized patients, respectively. Clinically relevant procedure-related complications (peritoneal laceration, hematoma) occurred in five patients.

**Conclusion:**

In summary, we demonstrate quantitatively that native nephrectomy prior to transplantation improved serum protein levels and anticipated post-transplant fluid intake needs in select children, reducing the risk of graft hypoperfusion and its postulated consequences for graft outcome.

## Introduction

Indications for native nephrectomy in patients with endstage renal disease (ESRD) prior to kidney transplantation (KT) are not well defined. Malfunctioning kidneys are removed if they are perceived to convey short- or long-term risks to the KT recipient or the graft. The spectrum of renal and urinary tract disorders leading to ESRD differs between adults and children. In addition, the removal of the native kidney(s) in young children and infants prior to transplantation entails specific clinical challenges related to fluid management and nutrition. Hence, indications for pre-transplant nephrectomy are expected to vary between the adult and pediatric patient groups. Studies on the biological effects of nephrectomy are limited, and outcome data are needed for informed decision-making.

The removal of endstage kidneys has been advocated for patients with large proteinuria, refractory hypertension, recurrent urinary tract infections (UTI) or urosepsis, urolithiasis, and polyuria [1, 2]. Heavy proteinuria and hypoalbuminemia are associated with an increased risk of thrombosis and thromboembolic events [3–5]. Disturbed hemostasis in nephrotic syndrome is thought to be caused by the loss of select coagulation factors in the urine, by unregulated protein and lipid production and by endothelial injury [3, 6–8]. Removing the native kidney(s) in nephrotic patients is expected to reduce the risk of acute graft thrombosis, intravascular volume depletion, nutritional deficit, and delayed wound healing [9].

There is minimal quantitative data on the effect of residual native kidney urine output and post-KT graft function in children [10, 11], and the few publications available on adults report conflicting results [12, 13]. Case reports [14] and anecdotal experience suggest that pediatric patients with polyuria due to tubular disorders or renal dysplasia may continue to have substantial native urine output for prolonged periods after transplantation. Large native urine output post-transplant can lead to extracellular volume depletion and hypoperfusion of the graft and may exacerbate the perfusion mismatch between small recipients and adult donors. Chronic graft hypoperfusion may be associated with an important—according to some studies, irreversible—drop in glomerular filtration rate (GFR) and accelerated fibrosis, particularly in the smallest renal transplant recipients [10, 15–19]. Volume depletion and renal hypoperfusion can exacerbate drug (e.g. cyclosporine) nephrotoxicity [20, 21] and further imperil the perfusion of adult-size grafts in young recipients. These considerations provide the rationale to optimize the conditions for post-transplant fluid management, including the removal of one or both kidneys.

In the absence of prospective data or evidence-based guidelines, we reviewed our pediatric experience of pre-transplant nephrectomies. The aims of this study were (1) to examine indications, surgical approach, and complications of native nephrectomies prior to transplantation and (2) to determine the effects of uni- and bilateral nephrectomy on selected biological and clinical parameters, specifically urine volume and protein loss (where applicable) and serum protein concentrations.

## Material and methods

### Study design

**Fig. 1:**
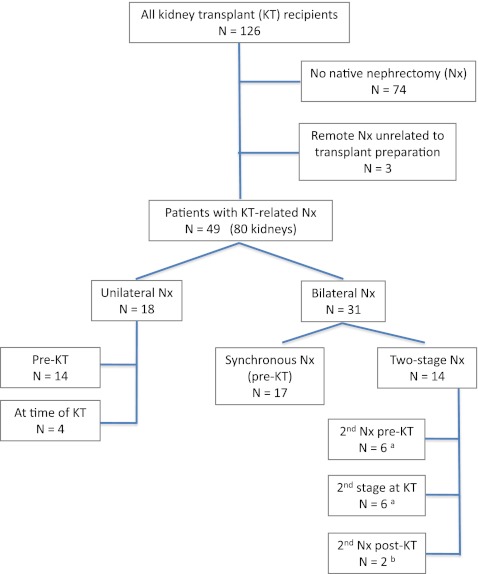
Summary of patients and nephrectomy procedures. ^a^One patient each had the first kidney removed previously for non-kidney transplant-related reasons, ^b^native kidneys removed 4 and 19 months post-kidney transplant (*KT*) because of frequent urinary tract infection

The study was designed as a single-center retrospective cohort study. We identified consecutive patients with uni- or bilateral native nephrectomies from a total of 126 consecutive graft recipients in our pediatric KT database. For reasons of data availability, we limited our review to patients who received a graft between December 1992 and October 2011 (18.9 years) (Fig. 1). The study was conducted in accordance with institutional ethics regulations; since it was a retrospective chart analysis, no informed consent was required. Demographic, clinical, and laboratory details, including serum creatinine, albumin, total protein and fibrinogen concentrations, UTIs and imaging, blood pressure, urine volume, and 24-h protein excretion pre- and post-surgery were extracted from charts and electronic records, including operation reports and pre- and post-operative clinic notes.

### Patients and surgical procedures

We identified 49 transplant recipients with uni- or bilateral nephrectomy (80 removed kidneys). Three patients who had undergone unilateral removal of poorly functioning kidneys due to lower urinary tract abnormalities at 4.7, 5.7, and 18 years prior to KT were excluded from the analysis (Fig. 1). With few exceptions [congenital nephrotic syndrome, Wilms tumor (WT), or Denys–Drash syndrome (DDS)], all patients had endstage renal disease at the time of pretransplant nephrectomy. Most patients were followed pre-operatively by the pediatric nephrology and (in part) the urology team at this institution. Ten patients have been described in a recent publication evaluating our experience with retroperitoneoscopic nephrectomy (RPN) in children receiving peritoneal dialysis [22]. The decision to remove one or both kidneys, concurrently or sequentially, depended on the diagnosis and the perceived benefit, such as preservation of some urine output during dialysis, and was based on consensus among the team of nephrologists and surgeons. Clinical judgment and psychosocial considerations, including the recipient’s age-related maturity, ability or willingness to cope with large volume fluid intake post-KT and overall medical adherence figured importantly as measurable (objective) parameters. The operative approach was at the discretion of the responsible surgeon.

### Clinical definitions

In the absence of a generally accepted definition, we specified *polyuria* for this review as a sustained urine output of >2.5 ml/kg/h. Proteinuria was defined as >4 mg urine protein/m^2^ per hour and *large (nephrotic range) proteinuria* as >40 mg/m^2^ per hour [23]. To assess the effect of (unilateral) kidney removal on urine output and proteinuria, we chose timed urine collection(s) closest to the procedure, generally <2 months pre- and post-nephrectomy. Measurements during the first 2 weeks post-nephrectomy were omitted from analysis to avoid confounding effects of perioperative fluid administration or dialysis adjustments. Charts were reviewed for the incidence of UTI at least 1 year prior to nephrectomy (median 2.8 years), between nephrectomy and KT, and ≥24 months after KT. Recurrent UTIs were defined as ≥2 documented infections within a 12-month period [24].

### Statistical analysis

Results are reported as median and range unless noted otherwise. Fisher’s exact test was used for categorical variables, and non-parametric (Wilcoxon matched-pairs signed-ranks and Mann–Whitney *U*) tests for continuous variables. Linear regression was used to assess longitudinal changes in urine output and biochemical results pre- and post-nephrectomy. Assumptions of a normal distribution of residuals, as well as a lack of heteroscedasticity, multicollinearity and non-linearity were satisfied (data not shown). A critical *p* value of 0.05 was used. The Bonferroni correction was applied in cases of multiple testing. Statistical analyses were performed using Stata v.10.1 (StataCorp, College Station, TX).

## Results

### Demographics, etiology of ESRD, and timing of nephrectomy

Of 126 pediatric renal transplant recipients who received a graft during the reviewed period, 49 patients (39%) underwent uni- or bilateral native nephrectomies (80 kidneys; Fig. 1). The median age at transplantation in the nephrectomized study cohort was 9.4 years (range 2.0–19.5 years). The main diagnoses leading to ESRD and nephrectomy were congenital (anatomical) anomalies of the kidney and urinary tract (CAKUT), nephropathic cystinosis, focal segmental glomerulosclerosis (FSGS), and congenital nephrotic syndrome (CNS) (Table 1). Histopathological reports were available for 21 CAKUT patients, with evidence of renal dysplasia in 71% of these.

**Table 1:** Nephrectomy cohort: clinical and demographic parameters

Etiology of ESRD	Number of patients (%)^a^	Age at Nx (years)^b^	Nephrectomies (Nx)^c^		Nx to KT^b^ (months)	Indication for Nx^d^
			Unilateral pre- /at KT	Bilateral synchronous/staged / at KT^e^		
CAKUT^f^	23/49 (46.9%)	6.0 (2.2–17.9)	6/3	5/3/6^g^	1.0 (0.0–25.5)^h^	UTI/VUR (*n* = 10), polyuria (8), proteinuria (1), polyuria and proteinuria (1), UTI and polyuria (1), UTI and proteinuria (1), hypertension (1)
Cystinosis	11 (22.4%)	11.1 (8.2–17.6)	5/1	5/0/0	1.1 (0.0–4.1)	Polyuria and proteinuria (6), polyuria (4), proteinuria (1)
FSGS	6 (12.2%)	8.1 (6.3–12.7)	2/0	3/1/0	17.4 (4.9–41.4)	Proteinuria (5), proteinuria and hypertension (1)
CNS	3 (6.1%)	4.8 (4.0–5.5)	–	2/1/0	2.3 (2.3–17.6)	Proteinuria (3)
Denys–Drash syndrome	2 (4.1%)	1.5, 4.2	–	1/1/0	1.5, 6.3	Wilms tumor risk, proteinuria (2)
ADPKD/TSC	1 (2.0%)	16.5	–	1/0/0	1.6	Angiomyolipoma, malignancy risk (1)
ARPKD	1 (2.0%)	2.8	–	0/0/1	0.0	Polycystic kidney (1)
Bartter syndrome	1 (2.0%)	15.6	–	0/1/0	0.39	Polyuria (1)
CIN	1 (2.0%)	15.8	1/0	–	7.1	Polyuria and proteinuria (1)

ADPKD, Autosomal dominant polycystic kidney disease; ARPKD, autosomal recessive PKD; CAKUT, congenital anomalies of kidney and urinary tract; CIN, chronic interstitial nephritis; CNS, congenital nephrotic syndrome; ESRD, endstage renal disease; FSGS, focal segmental glomerulosclerosis; KT, kidney transplant; Nx, nephrectomy; PUV, posterior urethral valves; TSC, tuberous sclerosis; UPJ, uretero-pelvic junction; UTI, urinary tract infections; VUR, vesico-ureteral reflux

^a^Total number of nephrectomized patients, *n* = 49

^b^Last nephrectomy in cases of staged procedures

^c^Data are presented as the number of patients

^d^Medical indications for procedure closest in time to the KT are given. Decision to operate was based on a combination of psychosocial and medical or laboratory factors. Number of patients for each indication are given in parenthesis. UTI/VUR (frequent UTI and/or high-grade VUR); proteinuria (large or nephrotic-range proteinuria)

^e^Second of staged Nx at time of transplant

^f^Specific diagnoses include: obstructive uropathy, *n* = 12 (7 with PUV, 4 with UPJ or lower urinary tract obstruction; 11/12 also with renal hypo/dysplasia); VUR, 8 (frequently with renal hypo-/dysplasia); cystic dysplasia, 2 (one with prune belly syndrome). 15/ 21 patients with CAKUT and available nephrectomy pathology had confirmed dysplasia

^g^Two patients with post-transplant native nephrectomy included

^h^Excluding one patient with remote first nephrectomy and post-KT second native nephrectomy

Thirty-four patients (69%) of the cohort commenced dialysis prior to, and 11 (23%) at the time of nephrectomy. Four patients (8%) were transplanted preemptively, before initiating dialysis: two of these were unilaterally nephrectomized 1–2 weeks prior to KT and two at transplantation. Of the patients with nephrectomy prior to KT, 51% received or intended to start peritoneal dialysis (PD), the remainder hemodialysis (HD).

Native nephrectomies were performed a median of 1.9 months (range 0–41.4 months) prior to transplantation (Table 1); 53% of the children had a living related donor graft, and the remainder received a graft from a deceased donor, almost exclusively adult.

### Indications for nephrectomy

Measurable parameters influencing the decision for native nephrectomy included polyuria, large proteinuria, and recurrent UTI. Of a total of 22 patients with polyuria as an indication for nephrectomy, 45% underwent unilateral nephrectomy, and the remainder underwent bilateral synchronous (18%) or staged nephrectomy (37%). The majority of patients with polyuria had CAKUT or cystinosis (45% each). The diagnoses associated with large proteinuria and nephrectomy (*n* = 22) were glomerular diseases (FSGS, CNS, and Denys–Drash syndrome; 50%), cystinosis (32%), CAKUT (14%), and chronic interstitial nephritis (4%). In this cohort, 82% of children with glomerular disease, 61% of those with CAKUT, and 45% with cystinosis underwent bilateral nephrectomy (Table 1).

### Surgical procedures

Eighteen patients (37%) underwent unilateral nephrectomy, 12 on the left side, this allowed for removal of the right kidney at the time of KT, if needed, which is the preferred side for the future graft. Two-thirds of the patients had both kidneys removed: 17 in a single (synchronous) operation, the remaining 14 sequentially. In ten patients the nephrectomy was performed at the time of transplantation, and two patients underwent native ureteronephrectomy 4 and 19 months post-transplant, respectively, for frequent UTIs attributed to the residual native unit (Fig. 1).

Of the patients for whom we had access to detailed surgical information, 31 organs were removed by minimally invasive surgery [transperitoneal, 5 kidneys; retroperitoneoscopic nephrectomy (RPN), 26 kidneys; total number of procedures, 24], and 36 by open surgery (transperitoneal, 7; extraperitoneal, 29; total number of procedures, 30). Thirty-eight percent of nephrectomies were combined with additional procedures, such as HD or PD catheter placement, cystoplasty or KT. Of the remaining patients, the median operation time was 3.0 h for unilateral and 7.2 h for synchronous bilateral procedures (*p* < 0.0001, Mann–Whitney *U* test). The duration of open versus minimally invasive surgery was 3.0 versus 5.2 h (*p* = 0.02). After stratification for the removal of one or both kidneys, the difference between open versus minimally invasive surgery time remained statistically significant for bilateral synchronous procedures (*p* = 0.002), but not for unilateral ones (*p* = 0.39, Mann–Whitney *U* test). These times reflect substantial trainee involvement.

### Effect of nephrectomy on urine output

The median daily urine output of 15 patients with polyuria who underwent unilateral nephrectomy (including the first of staged nephrectomies) decreased from 2.1 l (3.91 ml/kg/h) to 1.4 l (2.39 ml/kg/h; median change −40%; *p* < 0.005). The percentage post-operative reduction in urine output was not related to the amount of pre-operative polyuria (*p* = 0.73; linear regression *r* ^2^ = 0.30). Changes in urine output according to nephrectomy procedure are summarized in Table 2.

**Table 2:** Effect of nephrectomy (Nx) on urine output of polyuric patients^a^

Nephrectomy	Patients (*n*)^a^	Median urine output (range)^b^		
		Pre-Nx (ml/kg/h)	Post-Nx (ml/kg/h)	% Difference^c^
Unilateral	9	3.79 (1.43– .87)	2.22 (0.57–5.00)	−34* (−24 to −72)
Bilateral, first stage	6	4.08 (3.11–6.03)	2.39 (1.90–3.45)	−42* (−20 to −55)
Bilateral, second stage (rendered anuric)	7	2.87 (1.85–6.13 )	–	–
Bilateral, synchronous (rendered anuric)	4	7.62 (3.81–8.98)	–	–

*p < 0.05

–, Not applicable

CAKUT, congenital anomalies of the kidney and urinary tract

^a^Unilateral nephrectomy: CAKUT (*n* = 3 patients), cystinosis (5), chronic interstitial nephritis (1). Bilateral, staged nephrectomy: CAKUT (6), Bartter syndrome (1). Bilateral, synchronous nephrectomy: cystinosis (4). Unilateral nephrectomies at the time of KT were excluded. Some patients had additional indications leading to nephrectomy (see Material and methods)

^b^Urine output number reflects last documented collection prior to Nx. See Material and methods for polyuria definition. Decision to operate was based on a combination of psychosocial and medical factors

^c^Wilcoxon matched-pairs signed-rank test

**Fig. 2:**
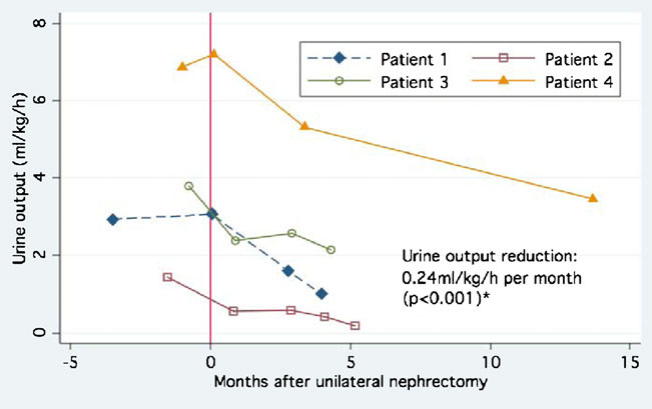
Gradual decrease of urine output following unilateral nephrectomy (in months; median 36%, range −10 to −70%). Depicted are all patients for whom data on serial, post-nephrectomy urine collections were available. *0* Time of nephrectomy. Subjects showed no appreciable decline of urine output following its stabilization after the initiation of dialysis 1–3 years prior to nephrectomy. *Asterisk* Analysis was performed with bivariate linear regression: effect of time on urine output (*r* ^2^ = 0.97)

We tested the possibility that urine output of ESRD patients further declined after unilateral nephrectomy towards the time of KT. Adequate urine collections were available from four patients covering an observation period of 4.0–13.7 months. Median urine output declined by 36% (range −10 to −70%), corresponding to a decrease of 0.24 ml/kg/h per month (Fig. 2). In contrast, pre-nephrectomy urine production in these subjects had been relatively stable over 1–3 years, after initiation of dialysis (results not shown).

### Effect of nephrectomy on proteinuria and plasma proteins

Twenty-two patients with large proteinuria (with or without polyuria or frequent UTI) underwent nephrectomy. After unilateral nephrectomy (including the first of sequential nephrectomies), urine protein excretion decreased by a median of 35%, from 3.7 to 2.4 g per 24 h (158 to 102 mg/m^2^/h) (*p* = 0.01, Wilcoxon signed-ranks test). Total serum protein, albumin, and fibrinogen concentrations were within reference ranges in 64, 69, and 36% of patients, respectively, prior to nephrectomy and increased modestly after the removal of one kidney, accompanied by a decrease of fibrinogen concentrations. Details are shown in Table 3.

**Table 3:** Effects of nephrectomy (Nx) on patients with large proteinuria^a^

Procedure	Variable and units^b^	Number of patients^c^	Pre-Nx^d^	Post-Nx	Improved pre-/post-Nx^d^	Percentage difference^e^
			Median (range)			
Unilateral nephrectomy	Proteinuria^a^ [<4 mg/m^2^/h]	9	157 (38–329)	100 (19–213)	0.11/0.13	−40* (−18 to −63)
	Serum albumin [31–48 g/l]	9	34 (20–41)	36 (29–41)	0.78/0.88	+6.0 (−3 to +75)
	Total serum protein [61–80 g/l]	9	63 (47–69)	67 (56–72)	0.67/0.63	+3.3 (−6 to +40)
	Plasma fibrinogen [1.5–4.0 g/l]	9	4.39 (3.20–7.95)	4.89 (3.02–6.57)	0.44/0.13	−1.3 (+28 to −49)
Bilateral nephrectomy (first stage)	Proteinuria [<4 mg/m^2^/h]	3	293 (79–305)	233 (36–280)	0.00/0.33	−21 (−8 to −54)
	Serum albumin [31–48 g/l]	4	23 (10–39)	22 (13–43)	0.50/0.50	+14 (−11 to +30)
	Total serum protein [61–80 g/l]	2	50 (37, 63)	56 (38, 74)	0.50/0.50	+10 (3–17)
	Plasma fibrinogen [1.5–4.0 g/l]	2	6.47 (3.74, 9.20)	5.45 (3.64, 7.26)	0.50/0.50	−12 (−3 to −21)
Bilateral nephrectomy (rendered anuric)^f^	Proteinuria [<4 mg/m^2^/h]	15	322 (100–659)	0	0.00/–	N/A
	Serum albumin [31–48 g/l]	15	25 (10–33)	36 (28–43)	0.13/0.93	+33** (3–260)
	Total serum protein [61–80 g/l]	15	51 (34–59)	62 (55–77)	0.00/0.73	+17** (7–52)
	Plasma fibrinogen [1.5–4.0 g/l]	14	6.15 (3.97–16.35)	3.99 (2.58–5.77)	0.07/0.55	−42** (−18 to −71)

*p < 0.05, **  < 0.005

N/A, not applicable

^a^Large proteinuria is defined as the excretion of >40 mg/m^2^ per hour (normal <4 mg/m^2^/h). In some cases, nephrectomy was performed for other indications than (large) proteinuria

^b^Variables reflect last documented measurements and urine collections prior to and at least 2 (up to 8) weeks post-Nx (see Material and methods). Some patients also presented polyuria or other conditions leading to nephrectomy (see Table 1). Decisions were based on a combination of psychosocial and medical factors, and previous laboratory data (preceding immediate pre-operative measurements). Reference ranges are given in square brackets

^c^Number of patients with available measurements for each biomarker

^d^Fraction of patients with urine protein excretion below the nephrotic threshold of 40 mg/m^2^/h, and with serum protein measurements within reference ranges, respectively, pre- and post-Nx

^e^Wilcoxon matched-pairs signed-rank test. Results are presented as the median, with the range in parenthesis

^f^Bilateral synchronous (*n* = 12) and staged bilateral nephrectomies (pre 1st/post 2nd stage measurements; *n* = 3)

**Fig. 3:**
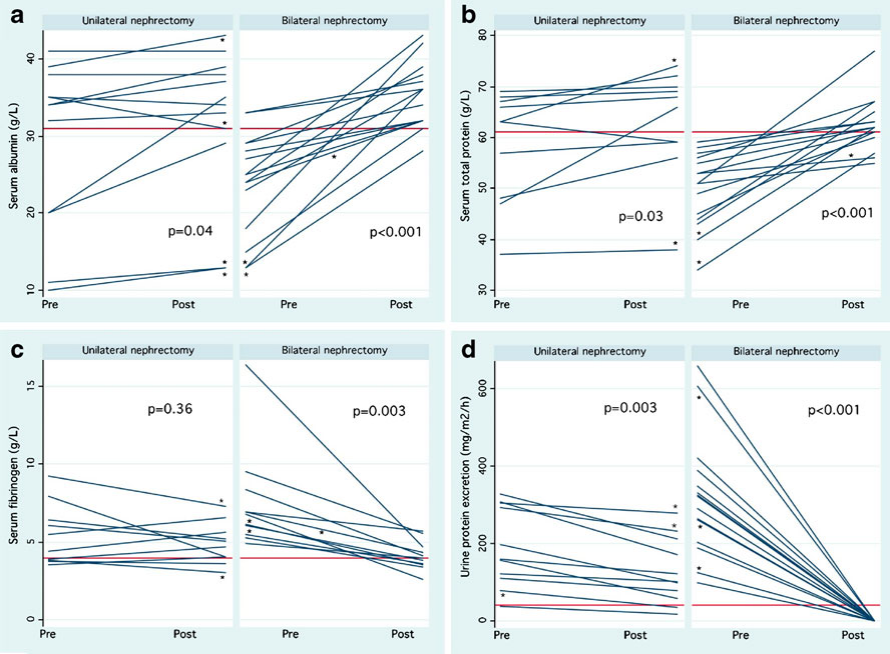
Serum albumin (**a**), total protein (**b**), and fibrinogen (**c**) concentrations, and urine protein excretion (**d**) pre- and post-unilateral and bilateral nephrectomy. *Horizontal* Lower reference ranges for serum tgroupalbumin and total protein (31 and 61 g/l, respectively), upper range for fibrinogen (4.0 g/L), and definition of nephrotic range proteinuria (40 mg/m^2^/h). Analyses were performed with Wilcoxon signed-rank test. In cases of sequential nephrectomies, an *asterisk* indicates measurements before/after the first (unilateral) and before/after the second procedure (bilateral nephrectomy). Note that the majority of patients undergoing unilateral nephrectomy had large proteinuria, but also polyuria or other risk factors leading to kidney removal

Patients scheduled for bilateral nephrectomy demonstrated greater pre-nephrectomy urine protein excretion than patients undergoing unilateral nephrectomy. Two of these patients also had polyuria. Unilaterally and bilaterally nephrectomized patients also differed with respect to their pre-operative serum albumin and protein levels (Fig. 3a, b). Within 2 months post surgery, initially low albumin and total serum protein concentrations in patients undergoing bilateral nephrectomy increased by 33 and 17%, respectively, while previously elevated fibrinogen concentrations decreased by 42% to almost normal levels (all *p* < 0.01) (Table 3).

Normal serum albumin, protein, and fibrinogen concentrations were recorded in 93, 73, and 55%, respectively, of bilaterally nephrectomized children with prior nephrotic range proteinuria, measured within 2 weeks to 2 months post-nephrectomy.

### Recurrent UTIs

To ascertain whether native nephrectomy led to the intended reduction of UTI rates, we analyzed nine patients who had undergone nephrectomy for recurrent UTI and for whom data were available (3 unilateral and 6 bilateral nephrectomies). Three of the patients were nephrectomized at or after KT, and for one of these patients there was insufficient post-operative information. Four of the five remaining patients had no more UTIs between nephrectomy and KT, and one had a single infection (observation period 1.1 years, range 0.1–2 years). Post-KT, three of the nine patients remained free of UTIs during 2 years of observation (range 0.3–4.8 years), and six patients had at least one recurrence of UTI (median 3.0 UTIs per person-year, range 0.2–24.4 UTIs). All six patients had persistent UTI risks, such as VACTERL sequence, posterior urethral valve bladder, cystoplasty, and intermittent catheterizations, not all of which are amenable to surgical correction. However, two of the latter patients underwent successful post-KT native nephrectomy, reducing the infection rate from 8.8 and 4.0 per year, respectively, to <0.34 per year.

### Hypertension

Persistent arterial hypertension (in addition to large proteinuria) was the indication for bilateral nephrectomy in a patient with FSGS. A second patient with dysplastic kidneys underwent bilateral renal embolization for hypertension and subsequently bilateral nephrectomy prior to his referral to our center. Post-nephrectomy blood pressure control improved in both patients and resulted in the reduction of the number of antihypertensive drugs from four to two and from two to zero, respectively.

### Nephrectomy-related complications

Clinically important, procedure-related complications were noted in five patients undergoing nephrectomy prior to KT for autosomal recessive polycystic kidney disease (ARPKD; *n* = 1 patient), DDS/WT (2), and cystinosis with polyuria or polyuria and large proteinuria (2). Peritoneal tears occurred in 4/20 “at-risk” patients, i.e., children receiving or planning to initiate PD, including 3/10 patients undergoing open retroperitoneal nephrectomy [*n* = 13 surgeries (removal of 14 kidneys); one associated with a large polycystic kidney and repair of bilateral hydroceles] and 1/10 patients undergoing bilateral synchronous RPN (11 surgeries, 14 kidneys). All four patients required temporary HD. The fifth patient experienced a retroperitoneal hematoma following open, extraperitoneal nephrectomy, which required transfusions of packed red blood cells and fresh plasma.

Minor complications related to the anesthesia or surgical technique were partial lung atelectasis (*n* = 3) and retroperitoneoscopy-associated (expected) transient subcutaneous emphysema (*n* = 2). Loss of urine output due to bilateral nephrectomy resulted in fluid overload and/or volume-mediated hypertension in five patients with ARPKD (*n* = 1) and FSGS (*n* = 4). They were managed medically by intensified dialysis.

## Discussion

Indications for peritransplant nephrectomy are controversial, and clinical practice varies according to perceived risks and benefits. The purpose of this study was to review our pediatric experience and to evaluate the biological effects of uni- and bilateral nephrectomy, specifically on urine output and proteinuria, where applicable, and serum proteins. We commonly base nephrectomy decisions on physiological, clinical and psychosocial determinants, with the goal to optimize the conditions for favorable graft outcome. Physiological risk factors are those compromising volume status and the perfusion of the (adult) graft in the pediatric recipient [10, 16, 19]. Clinical arguments include risks for graft and recipient due to UTI/urosepsis or persistent, severe arterial hypertension. Important psychosocial factors are the predicted inability or stated unwillingness to comply with increased post-transplant fluid intake, particularly among children and teenagers.

Our report represents the largest pediatric cohort examining clinical and biological effects of pre-transplant nephrectomies. Most prior publications have described indications and surgical outcomes in adult patients [25–30]. In addition to technical reports, we identified only two case series mentioning the inclusion of children: In the first study of 36 patients aged 10–67 years [28], eight nephrectomized patients were compared with 28 patients after ureteral reimplantation or no surgery prior to KT. The authors found no difference in the rate of UTIs between nephrectomized and non-nephrectomized recipients, but the numbers were small and children were not analyzed separately [28]. The second study [29] comprised 62 patients, including 11 children (14 nephrectomies). Pediatric indications were UTI (*n* = 9) or large proteinuria (*n* = 2), compared with hypertension, UTI, lithiasis, renal carcinoma, and polycystic kidney disease in the adult patients [29].

In our study, we did not intend to evaluate whether nephrectomy improves transplant outcome or graft and patient survival. The novelty of this report is that it describes the effects of native nephrectomy on urine volume, proteinuria, and serum proteins. Indeed, there are no data in the literature to quantify these biological effects. Our findings may help inform future decision-making. The renal diagnoses of the nephrectomized cohort are similar to those of contemporary, non-nephrectomized graft recipients transplanted in our center, with the exception of nephrotic syndrome patients. This further suggests that nephrectomy decisions were individualized, i.e. based on psychosocial factors and clinical judgment, and not (just) on the 24-h urine collection.

The decision to render young ESRD patients with preserved urine output anuric is not trivial. Where possible, the second (staged) nephrectomy was delayed to the time of KT to allow the advantage of residual urine production [31, 32] and to avoid additional surgeries and anesthesia. Removing the second kidney at the time of KT may circumvent this problem, but prolongs transplant surgery. It has been argued that patients may benefit from residual urine output and metabolic renal activity should they return to dialysis in case of early graft failure [33]. However, we did not encounter this scenario in our cohort.

Patients undergoing nephrectomy risk peritoneal laceration in addition to potential surgery and anesthesia complications. In our cohort, clinically important, procedure-related adverse events were documented in five patients, mainly peritoneal leaks that required temporary HD instead of the planned initiation or continuation of PD. Few patients experienced fluid overload and hypertension post-operatively, which is an expected outcome in patients rendered anuric. Interestingly, most of the latter patients had been nephrectomized for FSGS and persistent nephrotic syndrome. All responded well to medical management.

Compared to conventional, open nephrectomy, minimally invasive surgery shortened recovery and hospitalization times. Transperitoneal (open or laparoscopic) techniques would require switching from PD to (at least temporary) HD post-nephrectomy. The retroperitoneoscopic approach [34] preserved peritoneal integrity in 11 of 12 procedures. Three of ten patients undergoing open retroperitoneal nephrectomies suffered peritoneal laceration, but these procedures were all performed during the first half of the review period.

Our rationale for nephrectomy in children with polyuria was twofold: to reduce the risk of graft injury due to hypoperfusion and to alleviate the difficulty of maintaining high post-KT fluid intake. Hypoperfusion of the graft may have acute (tubular necrosis, graft thrombosis) and long-term consequences (accelerated chronic graft fibrosis) [9, 16, 19, 35].

Studying hemodynamic changes induced by the transplantation of adult-sized kidneys into young recipients, Salvatierra and his group reported that the blood flow in the transplanted renal artery diminished by one-half compared to the original measurements in the living donor and concluded that aggressive intravascular volume replacement is necessary to achieve and maintain optimal aortic (and renal arterial) blood flow following renal transplantation [16]. Berg et al. and others have shown that children receiving kidneys from pediatric donors revealed increasing absolute and stable relative GFR compared with those receiving adult kidneys [10, 18, 36]. By careful histological comparison of protocol biopsies, Naesen et al. [19] found functionally relevant, progressive non-immune graft injury in pediatric recipients soon after the transplant of adult-size kidneys. They postulated that graft ischemia associated with donor-recipient size discrepancy is a major risk factor for chronic tubulo-interstitial damage. The same group provided molecular evidence, employing cDNA microarray techniques, that early graft hypoperfusion induces the coordinated expression of profibrotic genes in the graft. Interestingly, gene activation was evident before any histological appearance of fibrosis [37]. These findings are relevant to our subjects who received almost exclusively adult donor kidneys.

Large proteinuria is believed to increase the risk of graft and extrarenal venous thrombosis due to the imbalance of pro- and antithrombotic factors [3, 5, 38], compounded by transplant-associated endothelial injury [39–41]. There is little doubt that persistent, severe nephrotic syndrome poses short- and long-term risks for the transplant recipient [40]. We have demonstrated that patients with compensated large proteinuria, but only mildly decreased serum albumin concentrations, experienced a modest rise of serum proteins after unilateral nephrectomy. Bilateral nephrectomy, on the other hand, fully corrected albumin, total protein and fibrinogen concentrations in the majority of previously hypoalbuminemic patients (see Fig. 3 and Results section).

It is of note that not all patients with recurrent UTI benefitted from pre-KT nephrectomy. This was likely due to variability in the underlying anatomical/developmental abnormalities affecting the lower urinary tract and bladder.

## Conclusion

The indication for pre-transplant native nephrectomy in our cohort was based on a combination of physiological, clinical, and psychosocial considerations. Unilateral nephrectomy reduced polyuria in all patients, by a median of 34%, and proteinuria by 40%, while bilateral (synchronous or staged) nephrectomy improved serum total protein, albumin and fibrinogen concentrations significantly by 17–42%. The main risk of the procedure is peritoneal laceration with temporary interruption of peritoneal dialysis. Proof that nephrectomy ameliorates graft fibrosis and long-term outcome in select patients would require a prospective, randomized, and controlled trial.
